# A Self-Provisioning Mechanism in OpenStack for IoT Devices

**DOI:** 10.3390/s16081306

**Published:** 2016-08-17

**Authors:** Antonio Solano, Raquel Dormido, Natividad Duro, Juan Miguel Sánchez

**Affiliations:** Departamento Informatica y Automatica, ETSI Informatica, UNED Juan del Rosal 16, 28040 Madrid, Spain; asolano2@alumno.uned.es (A.S.); nduro@dia.uned.es (N.D.); jsanchez210@alumno.uned.es (J.M.S.)

**Keywords:** Internet of Things, cloud computing, openstack, arduino

## Abstract

The aim of this paper is to introduce a plug-and-play mechanism for an Internet of Things (IoT) device to instantiate a Software as a Service (SaaS) application in a private cloud, built up with OpenStack. The SaaS application is the digital avatar of a physical object connected to Internet. As a proof of concept, a Vending Machine is retrofitted and connected to Internet with and Arduino Open Hardware device. Once the self-configuration mechanism is completed, it is possible to order a product from a mobile communication device.

## 1. Introduction

The Internet has evolved and grown beyond our expectations. It is expanding much more rapidly than it has done in the last decade. Internet of Things (IoT) is its new revolution, being one of the most relevant trends in the software industry. IoT is the fusion of the digital and physical world. In a world of IoT, billions of things or devices of all types and sizes are interconnected and uniquely identified. Devices are becoming instrumented, intelligent and interconnected. In this sense, maker and hobbies communities are hacking daily objects and connecting them to the Internet, discovering new ways to interact with them. For instance, how to retrofit a lamp and switching the lights from our smartphone is a simple case published in many blogs of such communities. Thanks to tiny embedded and cheap microcontrollers and sensors, it is not difficult to build up your own home automation solution. A relay and a WiFi or Bluetooth communication module below 5 USD, plus some lines of code borrowed from Open Hardware community would be enough to this end.

In this way, today our daily objects are becoming “smart”. This smarter and connected world has the potential to completely change our way of life. Examples of IoT solutions can be cars that talk each other about traffic congestion or medicine containers that remind the time to take your pills. In fact, clothing, factories … will eventually be “smart” as well. The possibilities are endless. By moving the logic devices uses to be embedded in electronics to the cloud it is possible leveraging cloud computing paradigms [1]. To this end, it is only required to connect our daily objects with low cost communication modules to Internet and to integrate the machinery of such objects with some sensors and actuators enabling the discovery of new ways to interact with them.

In just a couple of years a boom has occurred in the cloud based platforms to enable the IoT [2,3]. Early in 2009 Pachube [4] sets the foundations for such platforms and today there are hundreds of them enabling to collect data from our network of sensors and providing north-bound interfaces for data manipulation [5,6]. All of them claim to have plug-and-play mechanism to connect sensors and simple devices and they usually provide in-build simple scenarios such the mentioned example to control our retrofitted lamp. However, due to the intrinsic complexity of our physical world, in order to create digital version of complex objects or devices, which may be composed of many sensors and actuators [7], it is necessary to deploy bespoke logic at the application layer. In this context, to provide end to end self-configuration mechanisms is not an easy task.

The main challenge of this paper is to develop a simple plug-and-play mechanism to automate the deployment of digital version of complex objects in Internet, the so called in this paper digital avatars. These avatars are deployed following a model of Software as a Service (SaaS) in a cloud platform. In other words, the SaaS at the application cloud layer is the digital avatar of a physical object connected to Internet. To this end, a private cloud infrastructure with minimum hardware requirements using OpenStack [8] is deployed. OpenStack allows the creation of a very cost effective, flexible and elastic Information Technology (IT) infrastructure, taking full control of the resources and configuration required at the platform and the application layers. The key point of our work is to deploy a cloud-based plug-and-play mechanism for IoT devices in a simple way, with no need of performing ad-hoc and complex configuration actions by the cloud system administrator.

This plug-and-play mechanism and the cloud developed can be used by small, middle sized and large scale organizations with high efficiency and security. Multiple projects for multiple clients can be created in a cost efficiency way using this infrastructure.

As a proof of concept in this paper a vending machine to make it smarter is retrofitted. The evolution of the traditional architecture of buying in a vending machine by a cloud-based architecture is proposed. The core processes of the buying are offered through a SaaS business model. In this way, vending machines are connected and integrated in a cloud environment. It reinforces the concept of IoT by making objects smarter thanks to ubiquitous connectivity and new cloud computing paradigms [9]. This approach is achieved by moving business logic from real vending machines to the cloud. Usually, vending machines are owned and managed by vending operators. Therefore, vending machines are grouped and configured in a cloud multi-tenancy architecture where tenants are associated to vending operators and each tenant serves several vending machines. The open software used in the SaaS layer is OpenCart [10], a multistore shopping cart. This platform makes possible to offer service to many vending machines using a single domain. In this way there is no dependence on external Domain Name Server (DNS), apart from the public DNS where the domain is registered.

This paper is organized as follows. Section 2 briefly presents the cloud computing services and model. In Section 3, OpenStack architecture and components are described. The built of our private cloud and the plug-and-play automation are explained in Section 4. In Section 5 the model developed is explained and applied to retrofit a vending machine. Finally, some conclusions are presented in Section 6.

## 2. Cloud Computing at a Glance

Cloud computing is a modern computing paradigm that provides IT infrastructures. It involves deploying groups of remote servers and software network that allow the users to access different information from anywhere. The cloud computing removes the need for user to be in the same physical location as the hardware that stores data. The cloud provider can both own and house the hardware and software necessary to run home or business applications [11].

**Figure 1:**
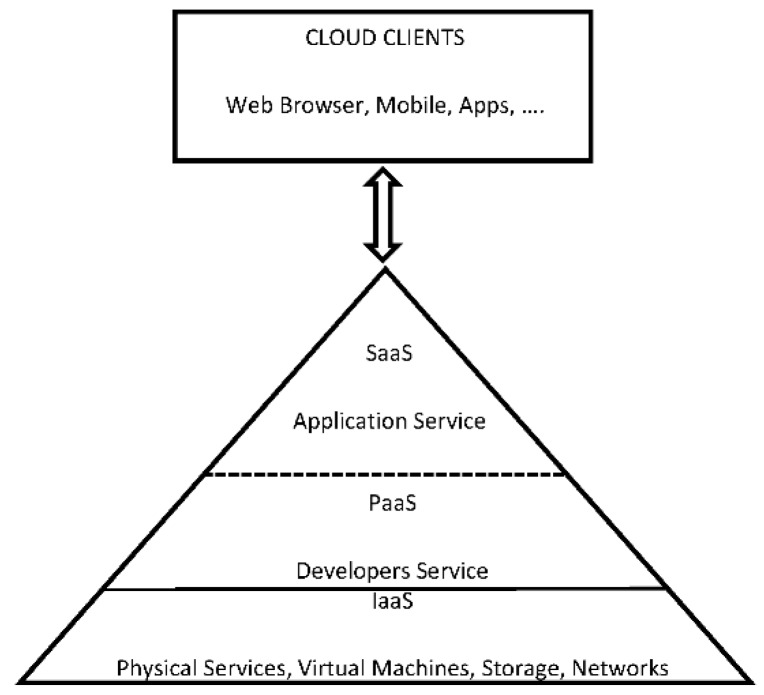
Cloud Service Models.

Cloud computing can be classified into three main categories attending to the service model it offers (see Figure 1):

- Infrastructure-as-a-Service (IaaS) is the most basic cloud service model. It provides virtual machines (VMs), load balancers, raw block storage, firewalls and networking services. Service provider owns the equipment and is responsible for housing, running and maintaining it.
- Platform-as-a-Service (PaaS) provides a computing platform including application program interfaces (APIs), operating system, development environments, programing languages execution environment and web servers. Users can access and use these tools to create applications on the service provider’s platform over the Internet.
- Software-as-a-Service (SaaS) offers users the hardware infrastructure, the software product and interrelates with the users through a portal. Cloud providers install and operate the application software in the cloud, authorizing an application to clients.

Cloud computing allows three deployment models: public, private or hybrid. If the services are provided over the Internet then it is public cloud, also called external cloud. When services are provided within an organization through intranet then it is a private or internal cloud. Hybrid cloud is an internal/external cloud, which allows a public cloud to interact with the clients but keep their data secured within a private cloud. 

## 3. OpenStack Overview

There are different free and open-source software solutions for setting up a private cloud [12]. Due to the simple, elastic, consistent and massively scalable services OpenStack offers, the proposed system is implemented using this software.

### 3.1. OpenStack Basic Architecture

OpenStack is able to control large pools of compute, storage and networking resources making use of a modular architecture, which uses different components to work together as a service. The three main components are the following:

1. OpenStack Identity Service. It provides identity, token, catalog of available services and policy. It tracks all OpenStack services installed.
2. OpenStack Compute Service. It is the cloud group controller. It provides a tool to deploy cloud including things like managing block storage, networking, computing resources, scheduling, authorization and hypervisors.
3. OpenStack Image Service. It is a mirror storage, query and retrieval system of virtual machines.

**Figure 2:**
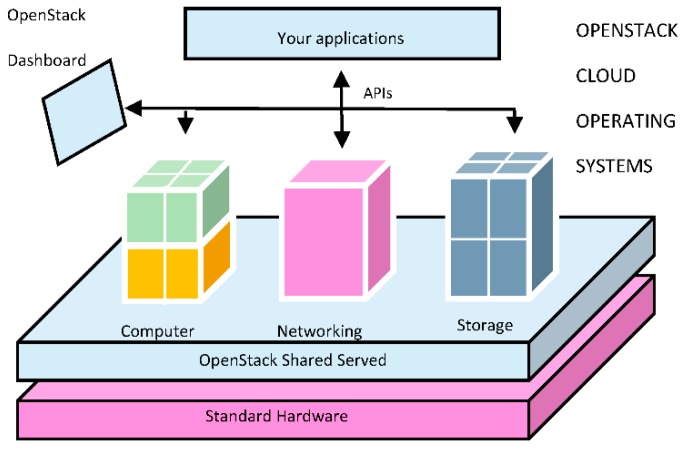
OpenStack Cloud Computing Operating System.

Figure 2 shows the architecture of the cloud operating system [8]. The OpenStack Storage Service shown in the figure is a highly scalable object storage system although it is not an essential component in the operated mode. It is worth to note that OpenStack allows the management of all the resources through a dashboard that gives administrators control while empowering their users to provision resources through a web interface.

Concrete implementation of each component in our development is shown in the next section.

### 3.2. Components of OpenStack

**Figure 3:**
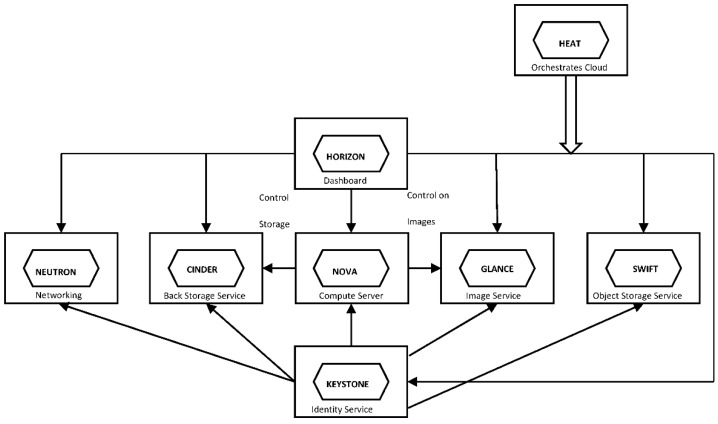
OpenStack system architecture.

OpenStack includes several key components such as Compute, Identity, Networking, Image, Block Storage, Object Storage, Telemetry, Orchestration, and Database. Figure 3 shows the OpenStack system architecture. A brief description of the different components and what they provide is given below.

- *KeyStone* provides a unified authentication and high level authorization service for all the components in the OpenStack family. It supports token based authentication.
- *Nova* is the computing controller for the OpenStack cloud. It is used to manage various compute resources, networking, authorization, and scalability needs of the OpenStack cloud.
- *Cinder* is a block storage component, which provides persistent block-level storage devices for use with OpenStack compute instances.
- *Glance* allows spinning up virtual machines quickly when users request them. Glance helps accomplish this by creating templates for virtual machines. It can copy or snapshot a virtual machine image and allow that to be recreated. Glance can also be used to back up existing images to save them and it integrates with Cinder to store the images.
- *Swift* is an object storage system for objects and files. Swift plays an important role in scalability.
- *Horizon* implements the dashboard. It allows the user to access cloud services platform by a web front-end interface. Things like manage instances and images, create keypairs or attach volumes to instances can be accomplished using it.
- *Neutron* is related with the networking. It enables tenants to create advanced virtual network topologies, improving performance and security.
- *Heat* implements an orchestration engine to launch multiple composite cloud applications based on templates in the form of text files.

## 4. Proposed General Architecture

### 4.1. The Challenge, Modeling Complex Digital Avatars

Similar to the Physical Web Google approach [13], each connected vending machine is identified by means of a Uniform Resource Locator (URL). The URL points to a Web application (Webapp), which is in fact the digital avatar of the vending machine. By accessing this URL from a smartphone, consumers will be able to interact with the vending machine and order products online. 

To create a digital avatar of a vending machine two facts have been considered: (i) a vending machine is an un-attendant point of sales; and (ii) nowadays, a point of sale on the Internet is an online shop. Therefore, the vending machine is modeled by means of open-source e-commerce software. However, to have a working online shop several steps are required. First, it is necessary to mirror the vending machine settings and product’s information such a price, stock … and then, to keep the vending machine and its digital avatar synchronized. Moreover, to reach the online shop, the URL has to be announced to consumers and obviously it has to be provided online payment mechanisms.

The challenge is how to streamline all these configuration steps with a simple plug-and-play mechanism. The proposed approach consists in building up an own private cloud to take full control of the deployment of virtual machines, which contains all the software and logic to become digital avatars of complex objects.

**Figure 4:**
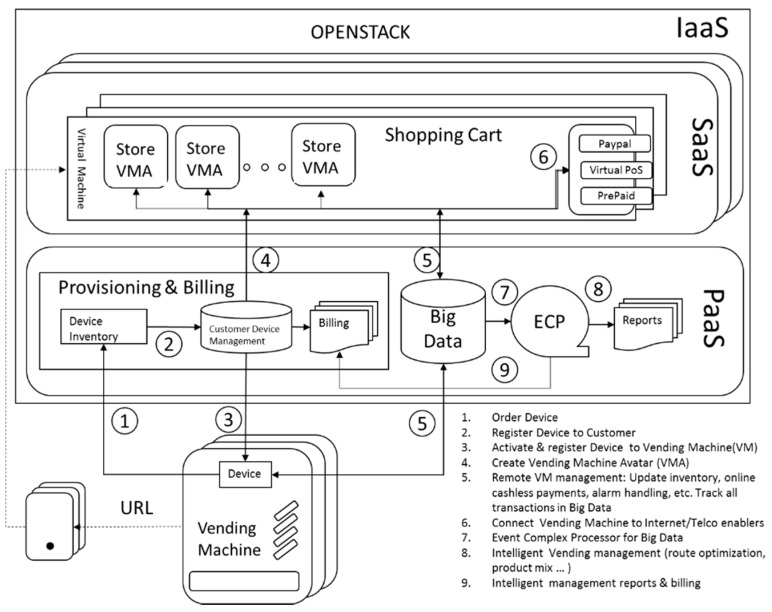
High level end to end system design applied to vending machines.

The Figure 4 shows the proposed high level system design applied to vending machines. It also describes the provisioning, management and billing flows to have a fully functional end to end solution. This paper focuses mainly on step 4: the instantiation of the vending machine avatar in the Cloud.

### 4.2. The Target Scenario

A vending operator buys a device to retrofit a vending machine. When the device is plugged into the vending machine, it initiates a self-configuration process consisting in:

1. Launching an instance of an online shop.
2. Reading the Telemetry of the vending machine to configure the online shop.
3. Publishing the URL of the online shop.
4. Buy online and dispense products onsite.

**Figure 5:**
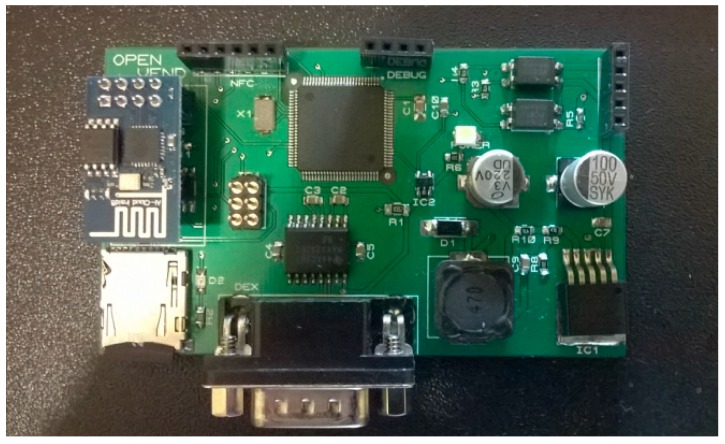
Arduino Mega compatible prototype.

As reference, Figure 5 shows one our bespoke Arduino Mega open hardware designs. This board is powered by the Mutlidrop Bus Standard (MDB) interface of the vending machine and it is able to communicate with our Cloud Solution via WiFi. The first time this board is powered on, it will initiate the plug-and-play mechanism describe on this paper. As outcome, we have published a demo [14] to show how to access to different vending machines and order products.

The self-provisioning process should last not more than 3–5 min.

### 4.3. System Architecture

**Figure 6:**
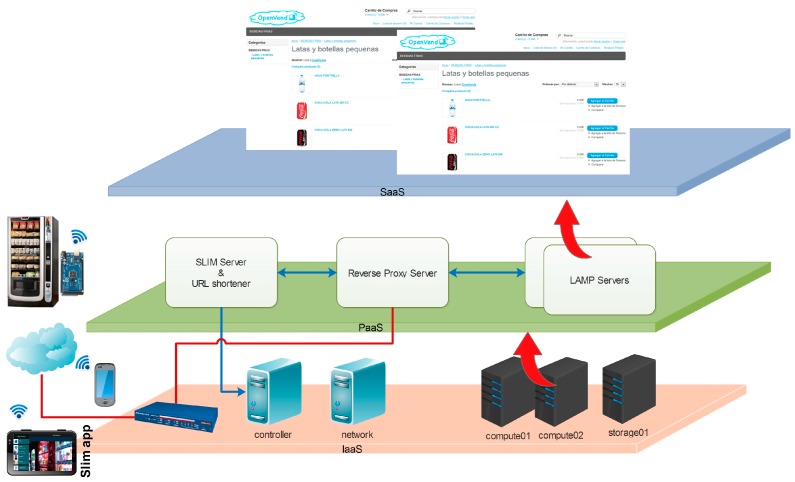
Building blocks of the proposed architecture.

Figure 6 shows a general block representation of the main components and interfaces that implement the global architecture of the system. In the Section 5 the mechanism of the plug-and-play with zero-configuration solution proposed is detailed.

The digital avatar of the vending machine resides in the SaaS layer. OpenCart is the free open software used to implement the e-commerce shopping cart. An OpenCart image is deployed for each vending operator. OpenCart is multistore, therefore each store inside OpenCart represents a vending machine.

The PaaS layer aims to facilitate the deployment of complex digital avatars such as our digital version of a vending machine in the Internet. The vending machine is connected to the Internet through an IoT module installed inside it. This module is built in an electronic board designed and assembled with low cost Arduino [15] compatible modules. The logical interfaces between the IoT module and the PaaS are based on REpresentational State Transfer (REST) technologies, commonly used in IoT deployments. Indeed, a RESTful API is an application program interface (API) that uses HTTP requests to GET, PUT, POST and DELETE data. The first time an IoT module is connected, the platform spins up a digital avatar of the vending machine in OpenCart’s multistore.

In the IaaS layer, among the different OpenStack components described in Section 3, NOVA is used to store and retrieve virtual disks (“images”) and associated metadata in GLANCE. The format chosen in GLANCE to store the actual virtual disk files in the Object Store is QEMU Copy-On-Write file (QCOW2), a flexible format, which allows images to grow on demand. Kernel-based Virtual Machine (KVM) is used for virtualization. KEYSTONE is the entry Service to the infrastructure, where all RESTful API queries from PaaS layer are received.

## 5. Implementing the Self-Provisioning Mechanism

OpenCart’s multistore mode, allows user to add more stores to the current installation by creating a subdomain structure for the stores, e.g., “http://store1.domain.com”, http://store2.domain.com” ... “http://storeN.domain.com”. However, this approach does not suit our needs. A subdomain, essentially, is an actual DNS entry. Therefore creating a subdomain is not necessarily so immediately obvious if our own DNS is not deployed. In addition, at times, even deploying our own DNS, the addition of your subdomain may not be immediately available due to potential DNS or Server-side propagation issues. In addition, from a SEO standpoint, it is difficult to increase rank in search engines and get traffic for N subdomains because Google treats them as different websites, regardless if they have one shared parent host.

To overcome the subdomain management issues it has been created a subfolder model for our OpenCart’s multistore. In this way, subfolders to address the digital avatars of our vending machines, e.g., “http://domain.com/store1”, “http://domain.com/store2” ... “http://.domain.com/storeN” are used. OpenCart documentation does not provide a full description of multistore, which may lead people to believe that subdomains are the only possible solution, but as it is shown below, it is possible. The following steps detail how to make a subfolder model working on OpenCart’s multistore:

1. Make a new Folder inside your OpenCart structure. Let’s call it “operatora001” because it will be the vending machine number “001” owned by “operator A”.
2. Go to the new folder titled “operatora001” and create an “.htaccess” file. Then copy all the strings from the original .htaccess file to it.
3. Add the following to the “.htaccess” file:
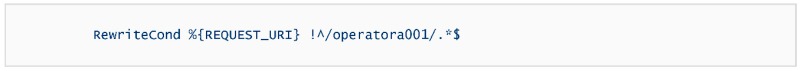
4. Create a new file inside the “*operatora001*” folder and name it “index.php.” The structure of this file is the following:
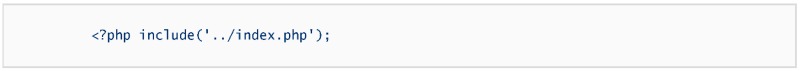
5. From the OpenCart admin panel go to Settings and create new Store. Add full URL path to the ‘Store URL’ of the sub-store like this: http://domain.com/*operatora001*/.

**Figure 7:**
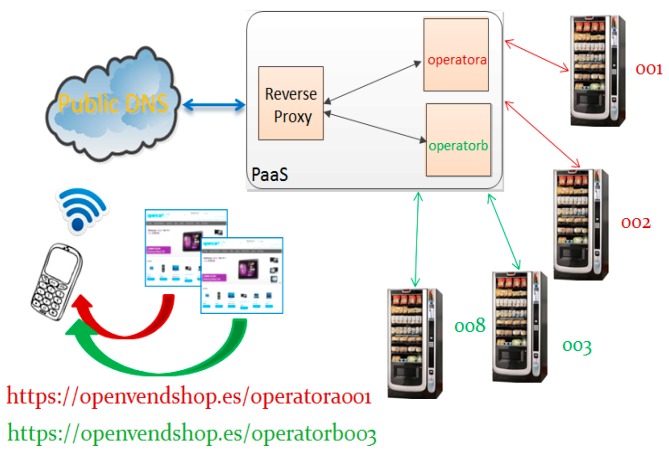
Access through domains.

At this point it is really important to note that different vending machines are accessed through only one registered domain, e.g., “openvendshop.es”, (see Figure 7). This mechanism allows not having to register domains for each vending operator, or for each vending machine. It makes the plug-and-play process independent of 3rd party DNS and it contributes to cost affordable solutions, as domain providers use to limit or charge for subdomains.

The automatic handling of the configuration files in the plug-and-play mechanism is detailed in Section 5.3 and Section 5.4. This is a complex task because of the mapping from subdomains to subfolders is performed in a reverse proxy which acts as the main entry point to the platform.

### 5.1. Underlying Reference Infrastructure

**Figure 8:**
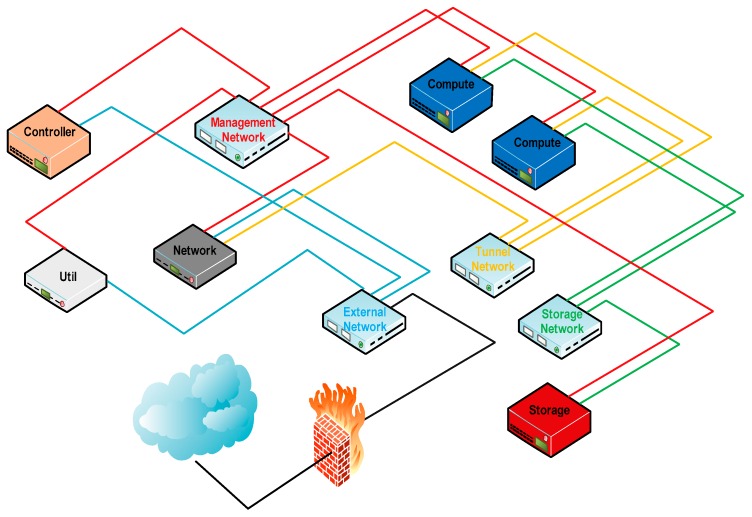
Typical OpenStack deployment.

Figure 8 shows a typical OpenStack deployment without High Availability (HA) used as reference for our project. The proposed design uses OpenStack Havana on Ubuntu 12.04 TLS. For this purpose the deployment consists of:

- one *controller node*, where services for the environment run.
- one *network node*, responsible for the virtual networking.
- two *compute nodes*, servers where Virtual Machines (VMs) are created.
- one *storage node* to store cinder volumes and images.
- one *util node* used to provide system administration functions, for monitoring and for maintenance purposes.

Regarding networking, four different networks are created and connected through switches. The usage of the networks is as follows:

- *external network*: it is a public network used for Internet access for all the nodes. Allows both inbound and outbound connections for VM’s.
- *management network*: used for communication between the controller and the compute nodes. It supports the internal communication between OpenStack components.
- *tunnel*: used for VM data communications.
- *storage*: used for communication between the storage nodes (cinder) and the compute nodes.

### 5.2. Automation of OpenCart Instantiation

Two of the main PaaS building blocks in Figure 6 are SLIM [16] and reverse proxy. SLIM is a PHP micro framework, which allows a quick deployment of RESTful APIs to communicate with the IoT modules based on Arduino open Hardware. Reverse proxy is based on Apache web server and is the entry point to the platform. It provides HTTPS for the webapps and RESTful APIs to IoT modules. Therefore, each time a new IoT module is plugged, SLIM initiates the plug-and-play process.

**Figure 9:**
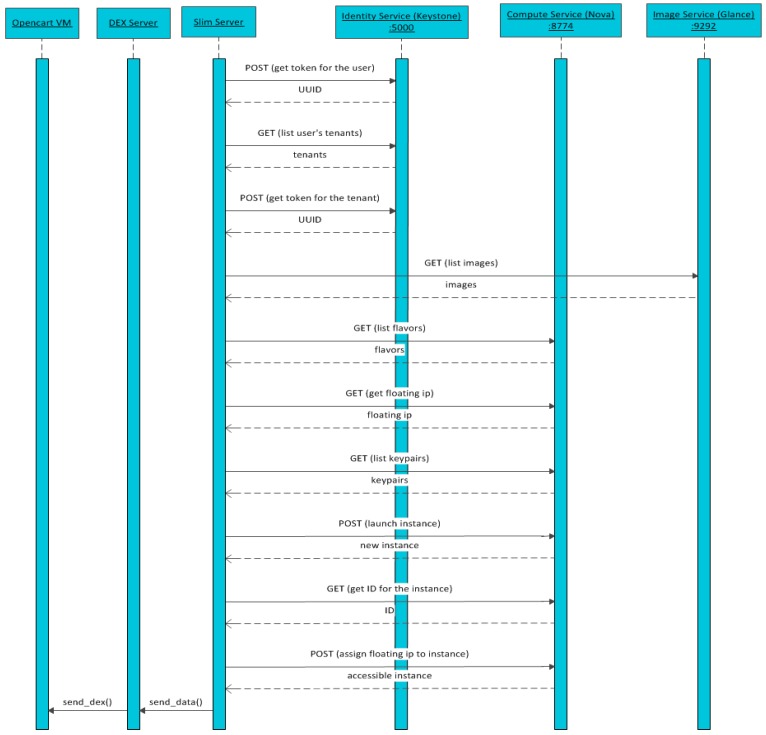
Sequence flow between SLIM and OpenStack’s components to instantiate OpenCart.

RESTful API queries are sent from the SLIM block to interact with the underlying IaaS controller node as Figure 9 shows. 

The main interactions result in:

1. Retrieving a Universally Unique IDentifier (UUID) token for subsequent secure interactions.
2. Retrieving an OpenCart image reference to be launched.
3. Retrieving “flavors” (number of virtual CPU’s, RAM, Disk capacity, Ephimeral Disk capacity)”, floating IPs and access keys used by new Virtual Machines instantiated in the PaaS.
4. Launch the new Virtual Machine (as a simple example of how to instantiate a virtual machine in OpenStack from an Arduino open hardware device, refer to source code provided in Appendix A).
5. VM is ready to receive telemetry data from vending machines to configure their digital avatars.

### 5.3. Self Configuration of OpenCart and Reverse PROXY

A self-configuration process is done by using shell scripts. The aim of the shell scripts is to automate the generation of VirtualHosts on the Apache servers and make some initial OpenCart configurations. These scripts are pre-programmed into the OpenCart and reverse proxy images and once a new instantiation is required from the SLIM server, their execution start. At this time, base64 encoded user data for the scripts are injected. Table 1 shows the OpenCart RESTful API specification to launch a virtual machine. 

The decrypted script injected in the value of the “user_data” field inside the body of the POST query looks as follows: 


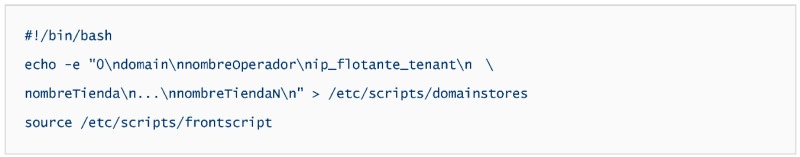


These data are mainly, the domain, the vending operator’s name and the number of vending machines in OpenCart’s multistore instance.

**Figure 10:**
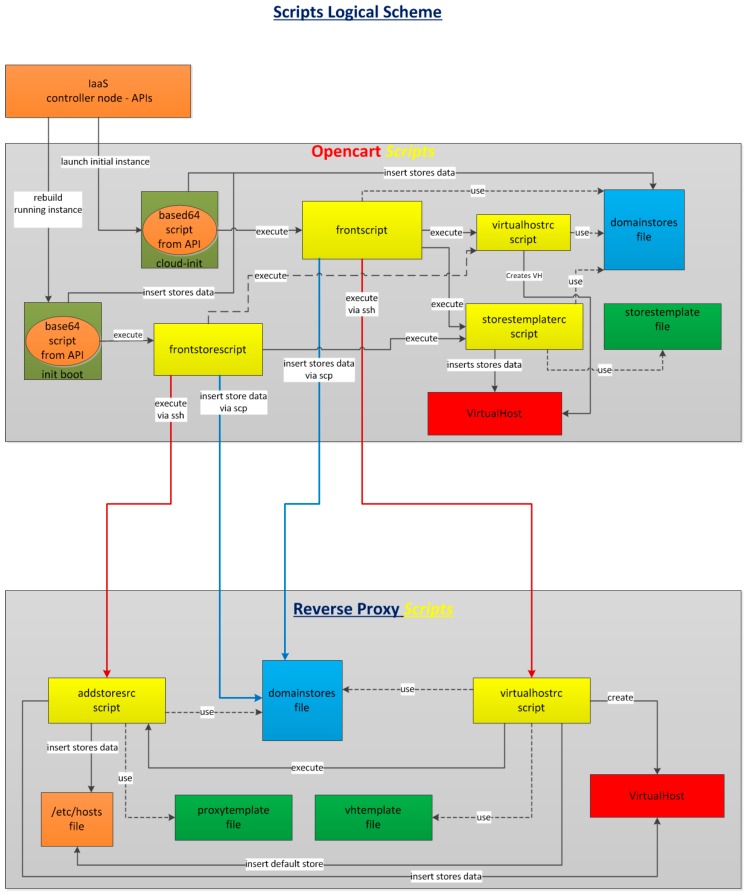
Scripts logical flow.

Figure 10 describes the scripts’ logical flow (see details in [17]). It can be noted that initially scripts are executed in the virtual machine where OpenCart is roll out, during instantiation time. From there, the execution flows to the reverse proxy. Once the cycle is finished, vending machines can be accessed from the Internet.

### 5.4. Shell Scripts Flow

This section presents an overview of the main interactions among scripts during the instantiation process.

The process starts with an OpenStack API request from SLIM block (Figure 9). Then the user data information is injected by an encoded base64 script (Table 1) that it is necessary in the rest of the process. Following this request, an automatic sequence of calls is triggered:

1. Initial script execution. It allows starting the logic to insert directives of Apache into the VirtualHosts. It also sends data to reverse proxy and execute scripts remotely.
2. VirtualHost creation on server where OpenCart resides. This script creates the VirtualHost for the domain if it does not exist.
3. Store data insertion into the VirtualHost. This script first includes injected data from OpenStack and then adds substitute directive into the VirtualHost. It also makes the necessary changes in the VirtualHost when a new default store is launched (see example in Appendix B).
4. Changes in OpenCart config.php file are also carried out. These scripts use some templates to fill the data. These are denoted in Figure 10 as Vhtemplate and Storestemplate.
5. Once the above process is completed, another similar process triggers in the reverse proxy, to configure an access from the Internet to OpenCart stores. The different scripts in this case accomplish the following actions:
  - VirtualHost creation for a particular domain to be used. This includes the creation of the default store into the VirtualHost, the addition of proxy directives for default store into VirtualHost, the addition of the domain and the IP address for the default store.
  - Insert stores into the VirtualHost to allow the access through Apache directives (see example in Appendix C).

These scripts also use some templates to fill the data (Vhtemplate and Proxytemplate).

### 5.5. Certification Authority (CA)

**Figure 11:**
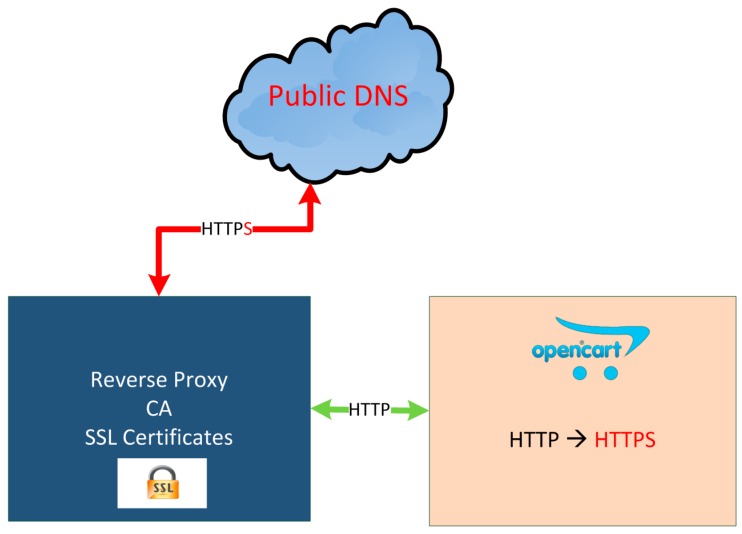
Https access.

As regards security, it is used SSL certificates signed by our own CA created inside the Reverse proxy, by means of a root certificate, avoiding external CAs. HTTPS between reverse proxy and Internet is provided (see Figure 11). For simplicity’s sake, the certificates are only retained in the reverse proxy, not affecting the security of the communications in the PaaS as they are performed through secured networks provided by OpenStack. 

For the creation of the certificates OpenSSL tools are used. Each domain has its own certificate.

## 6. Conclusions

The great potential of the Internet of Things (IoT) is widely known. To unlock its full potential in order to develop IoT solutions it is necessary to bring together connected devices and cloud computing.

Something like a universal plug-and-play to simplify programming and enable devices to be smarter is demanded from many forums. In this paper, a plug-and-play mechanism for IoT and apply it to retrofit a vending machine is presented. Open software like OpenStack, OpenCart, Arduino … has been used in the implementation in order to get an affordable solution in terms of cost issues.

Following our prototyping phase, the next step in this project would be to demonstrate the benefits of our approach in a real case scenario and integrate big data analytics such as the algorithms described at [18] to benefit of having a single entry point towards our cloud-based platform through a unique domain. A first approach of this work can be found in [19].

## Figures and Tables

**Table 1:** OpenCart RESTful API specification to launch a virtual machine.

**Method:** POST	**URL:** http://iaasopenstack.dyndns.org:8774/v2/{tenant_id}/servers
**Header Name**	**Value**
Content-Type	application/json
X-Auth-Token	<UUID>
**Body**	
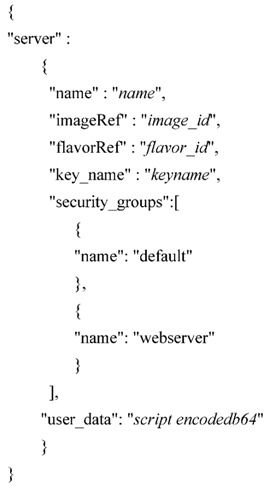	

## Appendix A

Below it is the source code for an Arduino Mega board using as communication module a General Packet Radio Service (GPRS) shield connected on serial port number 2 of Arduino Mega board. In this example, once the device is powered on, it instantiates a virtual machine in a private cloud based on OpenStack Havana (Ubuntu 12.04). As prerequisite it is needed to generate a Unique Universal Identifier (UUID) to access to OpenStack RESTful interfaces.

## Appendix B

As outcome of the plug-and-play mechanism, VirtuaHost are created in the folder /etc/apache2/sites-available/ of the virtual machine where OpenCart resides. An example is showed below.

## Appendix C

As outcome of the plug-and-play mechanism, VirtuaHost are created in the folder /etc/apache2/sites-available/ of the virtual machine where reverse proxy resides. An example is showed below.
